# *Trichoderma*-Synthesized Iron Oxide Nanoparticles Confer Vanadium Tolerance in Rice Through Multiple Defense Responses

**DOI:** 10.3390/ijms27219588

**Published:** 2026-10-27

**Authors:** Hafsah Shahbaz, Zaid Ulhassan, Zainab Shahbaz, Zohaib Kaleem, Aamir Mehmood Shah, Afrah E. Mohammed, Modhi O. Alotaibi, Dalal H. Alkhalifah, Ali El-Keblawy

**Affiliations:** 1 Institute of Animal and Dairy Sciences, University of Agriculture Faisalabad, Faisalabad 38040, Pakistan; hafsahshahbaz25@gmail.com; 2 School of Breeding and Multiplication (Sanya Institute of Breeding and Multiplication), Hainan University, Sanya 572000, China; 3 Key Laboratory of Forest Pathology, School of Forestry, Northeast Forestry University, Harbin 150040, China; zainab103@nefu.edu.cn; 4 Guangdong Provincial Key Laboratory for Plant Epigenetics, College of Life Sciences and Oceanography, Shenzhen University, Shenzhen 518000, China; naarzadazohaib6051@gmail.com; 5 College of Landscape Architecture, Sichuan Agricultural University, Chengdu 611130, China; aamirmehmood55@hotmail.com; 6 Research Department, Natural and Health Sciences Research Center, Princess Nourah bint Abdulrahman University, P.O. Box 84428, Riyadh 11671, Saudi Arabia; afamohammed@pnu.edu.sa; 7 Department of Biology, College of Science, Princess Nourah bint Abdulrahman University, P.O. Box 84428, Riyadh 11671, Saudi Arabia; mouotaebe@pnu.edu.sa (M.O.A.); dhalkalifah@pnu.edu.sa (D.H.A.); 8 Department of Applied Biology, College of Sciences, University of Sharjah, P.O. Box 27272 Sharjah, United Arab Emirates; akeblawy@sharjah.ac.ae; 9 Faculty of Pharmacy, Alsalam University, Tanta 31511, Egypt

**Keywords:** rice seedlings, vanadium toxicity, green nanoparticles, amino acids, oxidative stress

## Abstract

Vanadium (V) toxicity severely restricts plant growth and yield. The mechanistic basis by which *Trichoderma*-synthesized iron oxide nanoparticles (nFe) can improve V stress tolerance in rice remains unclear. To bridge this gap, we evaluated the protective efficacy of nFe in rice seedlings exposed to vanadium (V) stress. Results revealed that nFe lowered V-induced toxic impacts by minimizing V, MDA, H_2_O_2_, and O_2_^•−^ accumulation in leaves and roots. These reductions were correlated to improved photosynthesis phenomics, biomass, and membrane integrity, and reduced oxidative stress. Furthermore, nFe significantly increased the antioxidant enzyme activity and their gene expressions, and partially restored the amino acid (such as glycine, GABA, and proline) accumulation in V-treated seedlings, underscoring its crucial roles in plant defense. Additionally, nFe modulated the subcellular distribution of V by facilitating cell wall fixation and vacuolar sequestration by regulating chelator genes (*OsPCS1*, *OsMT2b*, *OsPCS2* and *OsGSH*). Moreover, nFe recovered V-induced cellular damages by preserving mesophyll structures, mainly chloroplast and thylakoids membrane. V accumulation, Fe uptake, photosynthesis, redox homeostasis and amino acid metabolism serve as key physiological indicators for evaluating crop cultivation under V-stressed conditions. Practically, the foliar application of biogenic nFe offers a promising and sustainable agricultural strategy to improve crop resilience and ensure food security in metal-contaminated soils.

## 1. Introduction

The contamination of agricultural soils by heavy metals (HMs) or metalloids has become a major environmental concern that exerts harmful effects on plant productivity and human health. Agricultural intensification, industrialization and urbanization have further exacerbated the global soil HM pollution [1]. Plants cultivated in these contaminated soils can easily accumulate toxic metal ions via their root systems, resulting in severe phytotoxic impacts on growth, development, and yield attributes [2,3]. To counteract HMs stress, plants perceive stress signals and trigger systemic defense strategies by modulating physio-biochemical and molecular status [4]. Various transcription factors or functional genes also participate in HMs, metalloid or nutrient stress responses or tolerance mechanisms [5,6,7,8,9]. The subcellular distribution of HMs influences the physiological response to metal toxicity and transporters involved in metal transport or detoxification [10]. Chelation of HMs in the cell wall and vacuolar sequestration are the major mechanisms of HM stress tolerance [11].

Among HMs, vanadium (V) is the fifth most abundant transition metal in the earth’s crust and the major contributions are from North America, Russia and China [12]. Intensified mining practices, anthropogenic actions and smelting activities have further increased its deposition in soil and water [13]. Globally, the average V concentration is 108 mg/kg, 87 mg/kg in Europe and 25 mg/kg in China [14,15]. It is easily bioavailable to agricultural plants and its potential effects are largely dependent upon the applied dose [16]. Low V levels may stimulate growth attributes but higher concentrations can impair normal growth and physiology [17]. Rice (*Oryza sativa* L.) is a dietary staple for more than 60% of people in China, signifying its vital role in agriculture [18], but it is markedly susceptible to toxic metals including V. Excessive V uptake via root systems severely stunted the plant’s growth traits, impaired chlorophyll synthesis, inhibited nutrition acquisition and caused overproduction of reactive oxygen species (ROS) in plant tissues [16]. The resulting oxidative damage further impacted the antioxidant defense system, increasing membrane permeability, lipid peroxidation and chromosomal damage [19,20,21]. In addition to this, V can enter the human body via the food chain through contaminated crops, raising potential health concerns [15,22]. Therefore, strategies that reduce V accumulation and toxicity in rice are important for sustaining crop productivity in V-contaminated environments.

Various stress-mitigators (biochar and micro-nutrients) have been evaluated to alleviate the V accumulation or toxicity in different crops. For instance, a recent study reported that Si at 1.5 mM effectively diminished the V accumulation in *O. sativa* tissues as well as improved the plant growth and biomass by minimizing oxidative stress (caused by ROS accumulation), increasing the expression of ROS-detoxification-related genes, improving photosynthesis and activities of antioxidant defense enzymes, and regulating activities of glyoxalase enzymes [21]. Another study demonstrated that phosphorus (P)-load biochar greatly minimized the V-induced toxic impacts in *O. sativa* [23]. Specifically, amendments with 3% P-loaded biochar improved the growth and biochemical attributes under V (60 mg/L)-stress conditions. Their findings revealed that P-loaded biochar significantly increased the plant fresh weight, SPAD chlorophyll, and antioxidant enzyme activities under V treatments. These outcomes suggest that P-loaded biochar can regulate biochemical pathways and facilitate biomass accumulation. However, further studies are required to elucidate the V-tolerance mechanisms.

Unlike conventional iron (Fe) fertilizer, Fe-nanoparticles (NPs) provide several benefits. For instance, their smaller size, tissue compatibility, and environmentally benign nature render them a prime agent for agricultural practices. Compared to chemically synthesized NPs, biologically synthesized NPs have displayed more efficacy to minimize the toxic impacts of abiotic stresses [24]. At present, there is limited information regarding the potency of biologically synthesized novel nFe (via foliar spray) in regulating V-stress tolerance in *O. sativa*. This research aims to clarify the effectiveness of novel nFe in curtailing V accumulation and relieving its inflicted phytotoxicity. The central assumption is that nFe may enhance V tolerance in *O. sativa* by regulating redox homeostasis and amino acid metabolism, modifying photosynthesis, the subcellular distribution and cell wall fixation capacity of V, and gene expression, and leading to ultrastructural changes. The studied biomarkers may offer a valued substitute for crop experts to boost *O. sativa* production in V (or other toxic metals)-polluted soils.

## 2. Results and Discussion

### 2.1. nFe Improved Seedling Growth and Biomass Tolerance Under V Stress

**Figure 1:**
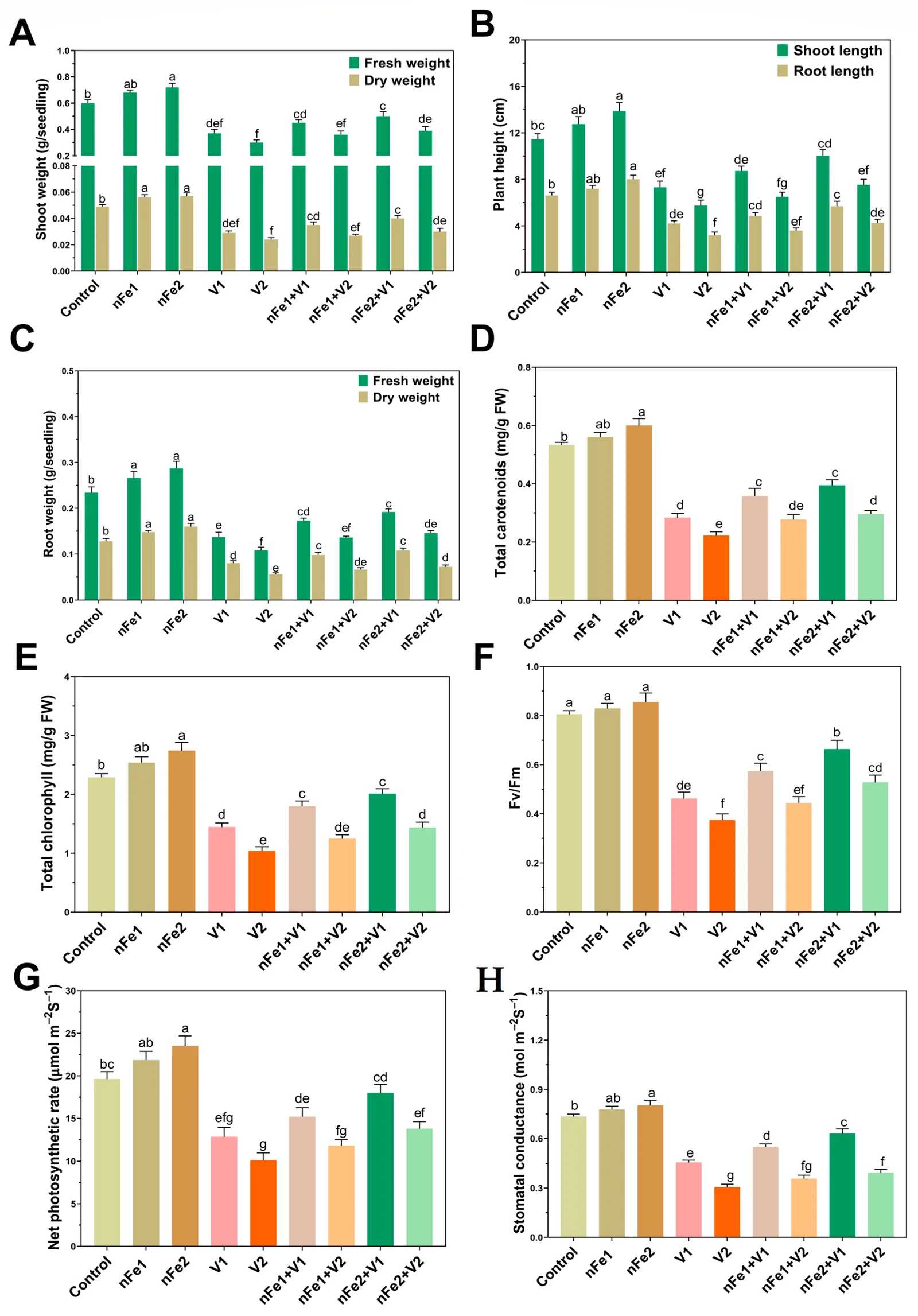
Effects of foliar-sprayed nFe on growth and biomass, leaf photosynthetic pigments, and gas exchange parameters of *O. sativa* plants with and without vanadium (V) stress: (**A**) shoot fresh and dry weight, (**B**) shoot and root length, (**C**) root fresh and dry weight, (**D**) total carotenoids, (**E**) total chlorophyll, (**F**) Fv/Fm, (**G**) net photosynthetic rate, and (**H**) stomatal conductance. Different lower letters in figures above the bars denote statistically significant differences among treatments (*p* < 0.05, two-way analysis of variance (ANOVA), followed by Tukey’s HSD test).

To examine the potential role of nFe in mitigating V toxicity, we first evaluated phenotypical responses of rice seedlings. Compared with control groups, exposure to V1 and V2 alone significantly decreased the root length (RL) by 38 and 54%, shoot length (SL) by 36 and 51%, shoot fresh weight (SFW) by 35 and 47%, and shoot dry weight (SDW) by 40 and 53%, respectively (Figure 1A–C). 

**Figure 2:**
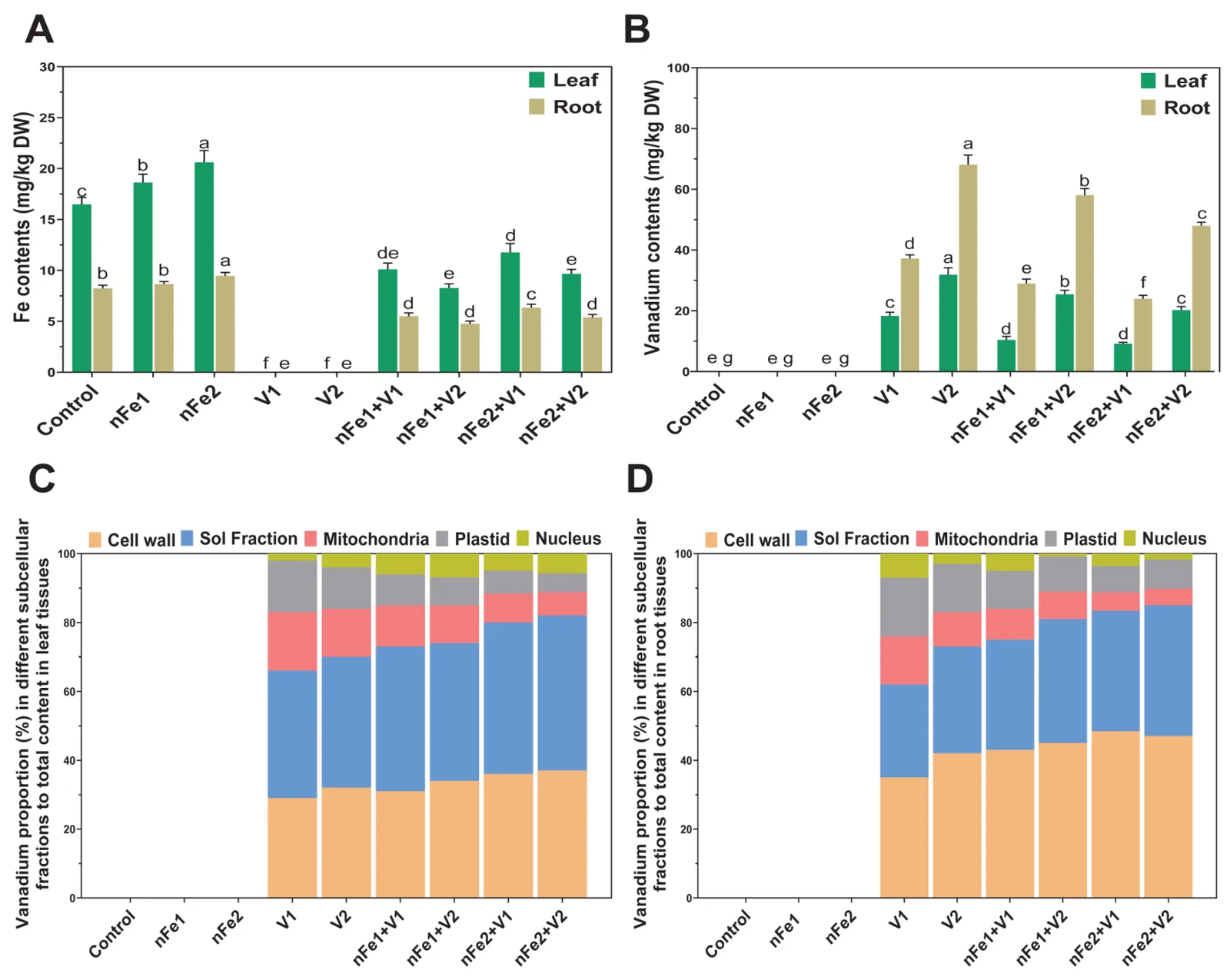
Effects of foliar-sprayed nFe on endogenous Fe and V contents, and subcellular distribution of V with and without vanadium (V) stress: (**A**) Fe contents, (**B**) V contents, (**C**) V proportion in different subcellular fractions to total V contents in leaves and (**D**) V proportion in different subcellular fractions to total V contents in roots. Different lower letters in figures above the bars denote statistically significant differences among treatments (*p* < 0.05, two-way analysis of variance (ANOVA), followed by Tukey’s HSD test).

**Figure 3:**
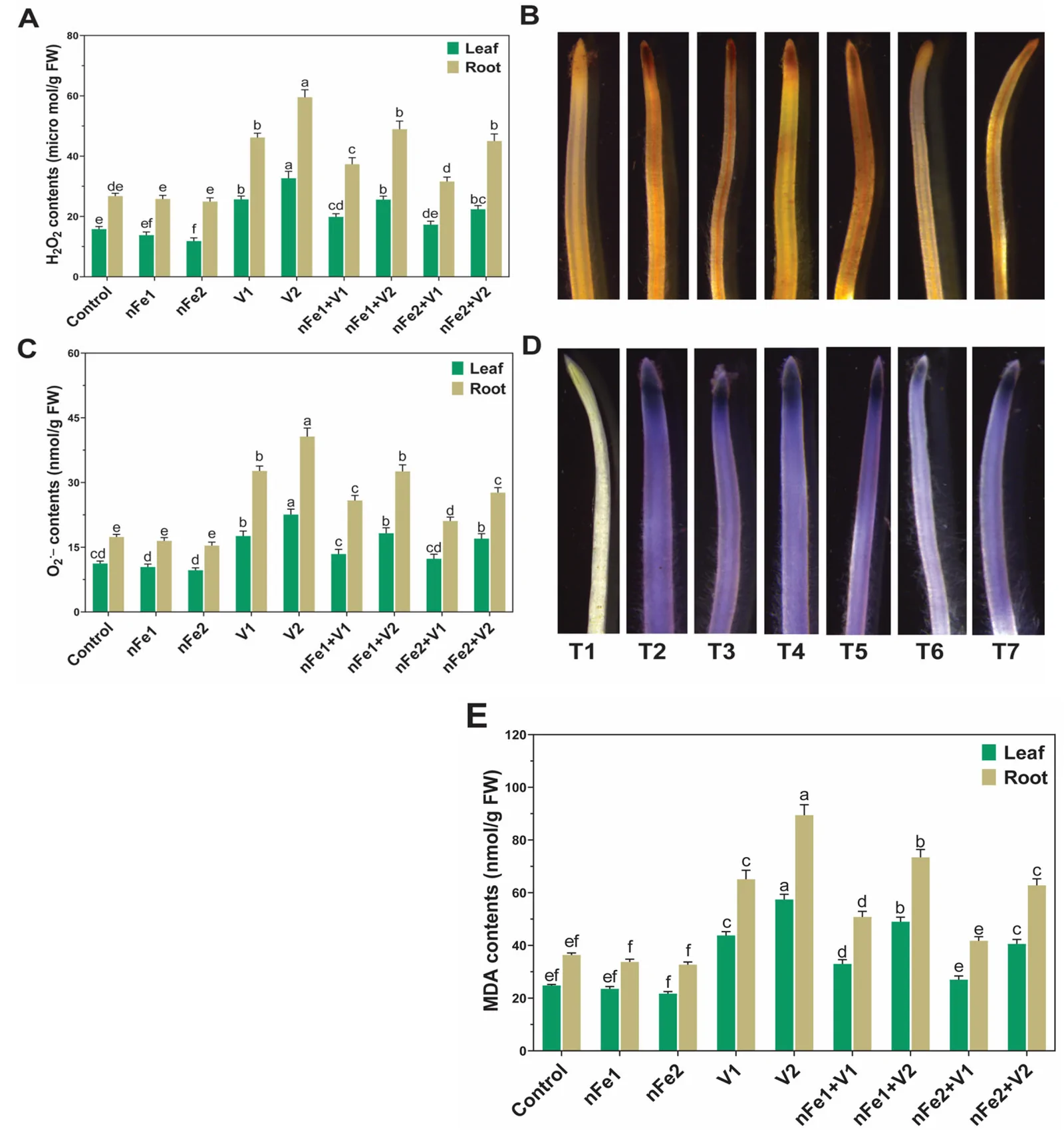
Effects of foliar-sprayed nFe on oxidative stress markers with and without vanadium (V) stress: (**A**) hydrogen peroxide (H_2_O_2_), (**B**) DAB root staining (**C**) superoxide (O_2_^•−^), (**D**) NBT root staining, and (**E**) lipid peroxidation as MDA. Different lower letters in figures above the bars denote statistically significant differences among treatments (*p* < 0.05, two-way analysis of variance (ANOVA), followed by Tukey’s HSD test).

**Figure 4:**
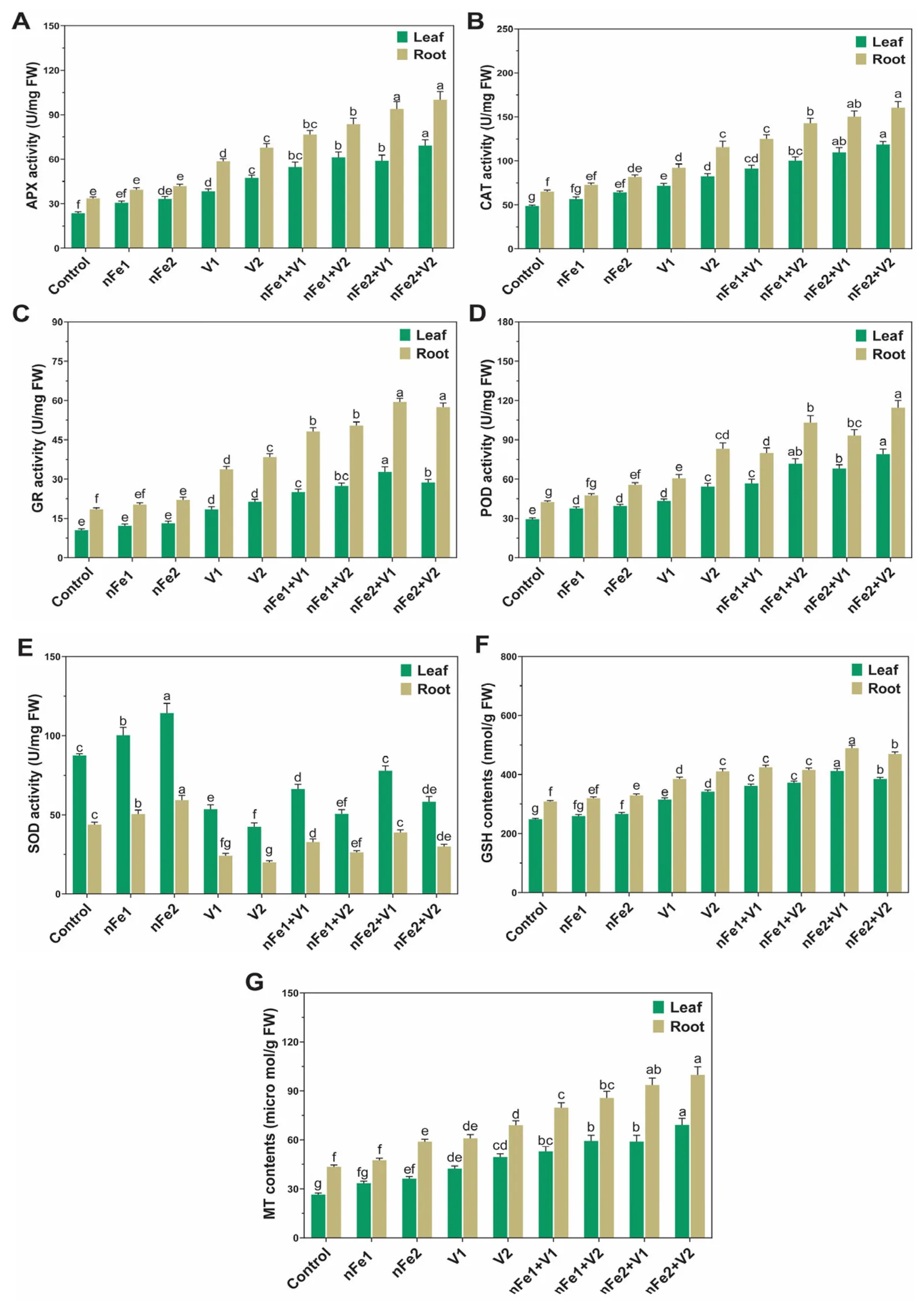
Effects of foliar-sprayed nFe on antioxidant enzyme activities, GSH and MT contents with and without vanadium (V) stress: (**A**) ascorbate peroxidase (APX) activity, (**B**) catalase (CAT) activity, (**C**) glutathione reductase (GR) activity, (**D**) peroxidase (POD) activity, (**E**) superoxide dismutase (SOD) activity, (**F**) glutathione (GSH) contents, and (**G**) metallothionein (MT) contents. Different lower letters in figures above the bars denote statistically significant differences among treatments (*p* < 0.05, two-way analysis of variance (ANOVA), followed by Tukey’s HSD test).

These findings displayed that V-only treatments, particularly V2, significantly (*p* < 0.05) reduced plant growth and biomass accumulation. The maximum suppression in root and shoot length as well as biomass production was directly correlated with excessive V accumulation in rice tissues (Figure 2B). Roots represent a major entry point of toxic heavy metals, where elevated bioavailability notably influences the metal bioaccumulation in plants [25]. The reduction in seedling length may therefore be due to the fact that V severely impaired root development, which leads to a restriction in the uptake of water or nutrient acquisition [26]. The direct cytotoxic impact of V on shoot tissues may also impair cellular metabolism and promote oxidative stress, thereby contributing to inhibited shoot growth [27]. This higher V accumulation impacts the physio-biochemical and cellular process such as through damage to cell functions, causing impairments in photosynthetic machinery, imbalanced nutrient assimilation, and ultimately, reduced growth and biomass as supported by earlier studies in *O. sativa* [21] and pepper [28]. The combined application (nFe + V), particularly nFe2 + V1 or V2, significantly diminished the toxic effects of V1 and V2 treatments as demonstrated by the higher RL (32 and 29%), SL (35 and 30%), SFW (33 and 28%), SDW (31 and 25%), RFW (34 and 31%), and RDW (30 and 26%) relative to V1 and V2 single-treatment groups, respectively. Results suggested that co-supply of nFe and V greatly minimized the negative effects of V on plant growth and biomass (Figure 1A–C). Under V stress, improved growth and biomass following nFe application were correlated with lower V accumulation (Figure 2B), higher Fe accumulation (Figure 2A), chlorophyll synthesis and photosynthetic activity (Figure 1D–H), lowered oxidative stress (Figure 3A–D), an enhanced antioxidant defense system (Figure 4A–D), and partial recovery of cell damage (Figure 8A–D). Possibly, NPs enhance cell division and root growth by establishing a physical barrier within plant tissues to mitigate HM stress [3] and ensure robust growth performance. Earlier studies have reported that different NPs can effectively improve the growth and biomass of *Z. mays* [29] and *C. sativus* [30] under V stress.

### 2.2. nFe Improved the Photosynthetic Phenomics Under V Stress

To better understand how nFe application regulates the photosynthetic apparatus under V stress, total chlorophylls, carotenoids, net photosynthetic rate (*Pn*), *Fv*/*Fm* and stomatal conductance (*g_s_*) were estimated. The results depicted that V (V1 and V2) stress caused a notable reduction in total chlorophylls (38 and 56%), carotenoids (46 and 57%), *Fv*/*Fm* (41 and 53%)*, Pn* (36 and 49%) and *g_s_* (39 and 54%), respectively, compared to relative control groups (Figure 1D–H). These findings exhibit that V stress severely impaired photosynthetic performance. The excessive V buildup may trigger a drop in photosynthetic pigment levels by inhibiting heme-related enzymes for chlorophyll synthesis (by restricting Fe-accumulation (Figure 2A) and disrupting chloroplast membranes and thylakoid structures, which can impair C assimilation as noticed previously in purple mustard [31] and pepper [28]). The combined application (nFe + V), particularly nFe2 + V1 and nFe2 + V2, significantly diminished the toxic effects of V1 and V2 treatments as indicated by improvements in total chlorophylls (40 and 36%) (Figure 1E) and carotenoids (38 and 32%) (Figure 1D), respectively. The improvement in photosynthetic pigments following nFe application can be linked to higher Fe bioavailability (Figure 2A), chlorophyll synthesis (Figure 1E), and their gene expression verification (Figure 7A), stabilized rates of respiration and net photosynthesis (Figure 1G,H), as supported by previous studies [32]. Vanadium stress can decrease the performance of photosystem II (PSII) photochemistry, as evidenced by a decline in *Fv*/*Fm* (Figure 1F). This reduced efficiency impedes overall photosynthetic rates (Figure 1G) and the transfer of electrons from PSII to PSI, ultimately hindering the synthesis of photosynthetic pigments [16]. The combined applications (nFe + V), particularly nFe2 + V1 and nFe2 + V2, significantly increase *Fv*/*Fm* contents (42 and 35%) (Figure 1F), respectively. This improvement suggests that nFe helped preserve PSII photochemical efficiency under V stress, consistent with earlier observations in mustard plants under V + melatonin treatment [20]. Furthermore, the decline in gas exchange parameters (*Pn* and *g_s_*) observed under V stress can be linked to impaired thylakoid membrane integrity and inhibition of pigment synthesis, resulting in reduced *Pn* and *g_s_* levels as observed in mustard [20,31].

The application of nFe2 + V1 and nFe2 + V2 notably increased the *Pn* (41 and 36%) (Figure 1G) and *g_s_* (39 and 31%) (Figure 1H), respectively. The increment in *Pn* and *g_s_* by nFe can be linked to higher photosynthetic and respiration rates that increased the synthesis of photosynthetic pigments as observed in *B. napus* under Cr + nSi treatments [3]. In comparison to V-only treatment, supplementing nFe significantly reversed the V-induced inhibitions in photosynthetic parameters. These positive effects in photosynthesis were attributed to increased leaf Fe accumulation, enhanced respiration rates, reduced oxidative stress and improved light-harvesting capacity.

### 2.3. Exogenous nFe Increased Endogenous Fe and Regulated V Accumulation and Its Subcellular Distribution Under V Stress

To examine how nFe influences the accumulation of V and Fe, we evaluated their endogenous synthesis in leaves and roots (Figure 2A,B).

The results showed that V-only treatment significantly increased the endogenous V1 (18.3 ± 1.2 mg/kg) and V2 (31.8 ± 2.1 mg/kg) contents in leaves and V1 (37.2 ± 1.3 mg/kg) and V2 (68.07 ± 3.4 mg/kg) contents in roots relative to control treatments (Figure 2A,B). The higher V accumulation in roots than leaves reflects that roots effectively retain V, limiting its translocation to above-ground plant parts. In contrast, the application of nFe considerably decreased the V accumulation in leaves (23–38%) and roots (19–29%) (Figure 2A,B). The “nano-effect” of nFe may have contributed to reduce the V accumulation by inhibiting its root to leaf transport, as reported in previous studies [3,32]. The treatments of only nFe, nFe1 and nFe2 considerably enhanced endogenous Fe levels by 17 and 26% in leaves, and 11 and 17% in roots under V stress (Figure 2A). This suggested the involvement of nFe in boosting the defense system and inhibiting V accumulation. But, V-only treatments greatly minimized internal Fe contents in plant tissues. These findings strongly suggest that nFe reduced the plant V accumulation in association with increased endogenous Fe contents under V-stress conditions. Prior research has demonstrated the effectiveness of melatonin and modified biochar in lowering V accumulation in different plant species [16,23,31].

Analysis of V subcellular distribution in leaves and roots demonstrated distinct partitioning patterns in response to nFe treatment (Figure 2C,D). V was mainly accumulated in the cell wall in roots and soluble fraction in leaves, followed by mitochondrial and plastid locations, with a smaller proportion in the nucleus of both leaf and root tissues. These reports were supported by earlier findings that V was mainly deposited in the root cell wall [33]. The incorporation of nFe significantly increased the V fixation in root cell wall and resulted in higher proportions of V in soluble fractions compared with V-stressed plants without nFe. These reports suggest that nFe may alter V partitioning by increasing its association with the cell wall and soluble fraction, thereby minimizing its distribution to cellular components via enhanced vacuolar sequestration. Negatively charged cell wall components may facilitate sites for V binding and limit its translocation to other cellular organelles. The soluble fraction was another major compartment containing V fractions. Generally, toxic metal ions can bind to cellular cytosolic ligands and convert them to less toxic complexes, a process known as chelation [34]. The findings from the current study indicate that nFe regulated V subcellular distribution by restoring safe compartmentalization and V chelation to mitigate V toxicity. Earlier studies corroborated our findings that nFe enhanced Cd fixation to the cell wall and vacuole compartmentalization and inhibited entry to cellular organelles in *B. napus* tissues [34].

### 2.4. nFe Mitigated Oxidative Damage and Maintained Membrane Integrity Under V Stress

To evaluate the impacts of nFe on modulating the oxidative damage along with cell membrane integrity under V stress, we quantified lipid peroxidation as malondialdehyde (MDA), hydrogen peroxide (H_2_O_2_), and superoxide (O_2_^•−^) in leaves and roots (Figure 3A–E).

Results showed that V (V1 and V2)-only treatments exhibited a notable increase in the accumulation of MDA (78 and 123%/87 and 137%), H_2_O_2_ (67 and 109%/79 and 118%), and O_2_^•−^ (58 and 107%/88 and 129%) in leaves/roots, respectively, in comparison to their relative controls. The data revealed that V induced significantly greater accumulation of oxidative damage indicators in roots than leaves, highlighting that oxidative stress was more pronounced in roots. The current results suggest that elevated V accumulation in rice tissues was likely associated with significant oxidative damage, as indicated by excessive ROS generation and increased MDA content. Vanadium may interact with the electron transport chain, leading to increased production of ROS. This elevated ROS generation can cause lipid peroxidation, as indicated by increased MDA, ultimately disrupting membrane stability and damaging cell structures as noticed in earlier studies [16,31,35].

Conversely, exposure to nFe (nFe1 and nFe2) treatment substantially lowered the MDA (19–38% and 21–32%), H_2_O_2_ (22–34% and 19–32%) and O_2_^•−^ (19–31% and 22–36%) contents in leaves and roots, respectively, in V-stressed plants (Figure 3A,C,E). The current findings suggest that nFe mitigated the oxidative stress and lipid peroxidation helped maintain cellular membrane integrity by curbing excessive production of ROS and MDA. These results indicate that nFe ameliorated V-induced oxidative damage. Previous studies have shown that NPs and/or phytohormones can effectively mitigate HMs or V-induced oxidative damage and support cellular membrane integrity in *O. sativa* [31,36] and *B. napus* [3,34]. To corroborate the quantification of H_2_O_2_ and O_2_^•−^ and their regulation by nFe, rice root samples were stained with 3,3-diaminobenzidine (DAB) and nitro blue tetrazolium (NBT) (Figure 3B,D). Compare to non-stressed control treatment, root samples subjected to V-only treatments exhibited intense dark brown (H_2_O_2_) and dark blue (O_2_^•−^) staining. In contrast, nFe treatment prominently lowered the severity of DAB and NBT staining in V-exposed roots (Figure 3B,D). This supports the role of nFe in mitigating V-induced oxidative damage by attenuating the free radical overproduction.

Conversely, exposure to nFe (nFe1 and nFe2) treatment substantially lowered the MDA (19–38% and 21–32%), H_2_O_2_ (22–34% and 19–32%) and O_2_^•−^ (19–31% and 22–36%) contents in leaves and roots, respectively, in V-stressed plants (Figure 3A,C,E). The current findings suggest that nFe mitigated the oxidative stress and lipid peroxidation helped maintain cellular membrane integrity by curbing excessive production of ROS and MDA. These results indicate that nFe ameliorated V-induced oxidative damage. Previous studies have shown that NPs and/or phytohormones can effectively mitigate HMs or V-induced oxidative damage and support cellular membrane integrity in *O. sativa* [31,36] and *B. napus* [3,34]. To corroborate the quantification of H_2_O_2_ and O_2_^•−^ and their regulation by nFe, rice root samples were stained with 3,3-diaminobenzidine (DAB) and nitro blue tetrazolium (NBT) (Figure 3B,D). Compare to non-stressed control treatment, root samples subjected to V-only treatments exhibited intense dark brown (H_2_O_2_) and dark blue (O_2_^•−^) staining. In contrast, nFe treatment prominently lowered the severity of DAB and NBT staining in V-exposed roots (Figure 3B,D). This supports the role of nFe in mitigating V-induced oxidative damage by attenuating the free radical overproduction.

### 2.5. nFe Regulated Antioxidant Defense and Metal Chelator (Glutathione and Metallothionein) Accumulation Under V Stress

To further investigate how nFe mitigated V-induced oxidative damage and improved plant defense, we assessed the activities of different antioxidant enzymes (Figure 4A–E) together with the expression of relevant genes (Figure 7A,B).

Our results showed that V stress augmented the activities (and related gene expression) of antioxidant enzymes, including CAT (*OsCAT*), APX (*OsAPX*), POD (*OsPOD*) and GR (*OsGR*) but notably decreased the SOD (*OsSOD*) activity in leaves or roots. Compared with the corresponding V-only treatment, a progressive up-regulation of the activities and expression of genes related to POD, CAT, APX and GR enzymes, together with a reversal of the response of SOD activity and *OsSOD* expression, was found in leaves and roots following the nFe + V treatments. The enhanced activity of antioxidant enzymes and their transcript levels with nFe treatment was attributed to reduced ROS production. These results indicate that the application of nFe effectively participates in antioxidant defense mechanisms, leading to a significant reduction in V-induced oxidative stress by scavenging ROS. The induction in antioxidant enzyme activities under nFe + V treatments supports the enhanced *O. sativa* tolerance to V stress. Previous studies have shown that exogenous application of nFe can enhance antioxidant enzyme activity in plants under abiotic stress conditions [32,34]. The application of different plant growth regulators was reported to induce antioxidant enzyme activity in *O. sativa* [16,21,36] under HMs stress. The antioxidant enzyme response to nFe is intricate and can change depending on the targeted plant, exposure technique, and extent of HM stress.

Compared to non-stress control conditions, GSH and MT contents were elevated in both leaves/roots by 29/25%, and 41/34%, and 59/43%, and 72/57% in leaves/roots in V1- and V2-stress conditions, respectively. The addition of nFe, especially nFe2 + V1 and nFe2 + V12, further increased the GSH and MT contents by 33/27%, and 14/17%, and 39/52%, and 40/48% in leaves/roots compared to relative V1 and V2 treatments, respectively (Figure 4F,G). Metal chelators such as GSH and MT play crucial roles in reducing the inter-cellular toxicity of toxic metal ions, facilitating metal sequestration and regulating metal-tolerance mechanisms. Under V stress, the strong induction of GSH and MT in plant tissues by nFe may contribute to V tolerance via metal chelation and antioxidant defense, as explained in [37]. Plants had no other option but to induce GSH and MT to chelate V and then sequester it into less-sensitive cellular organelles, thereby reducing its cellular toxicity. Gene expression analysis verified the involvement of nFe in up-regulating the expression of *OsPCS2* (phytochelatin-related), *OsGSH* and *OsMT2b* genes in plant tissues, which was attributable to enhanced metal chelation and sequestration responses. An earlier study reported that nano-zinc induced the levels of GSH, MT and PCS and up-regulated their transcripts, and largely participates in Cd sequestration, reducing cellular localization and enhancing Cd tolerance [37].

### 2.6. nFe Regulated Amino Acid Metabolism Under V Stress

**Figure 5:**
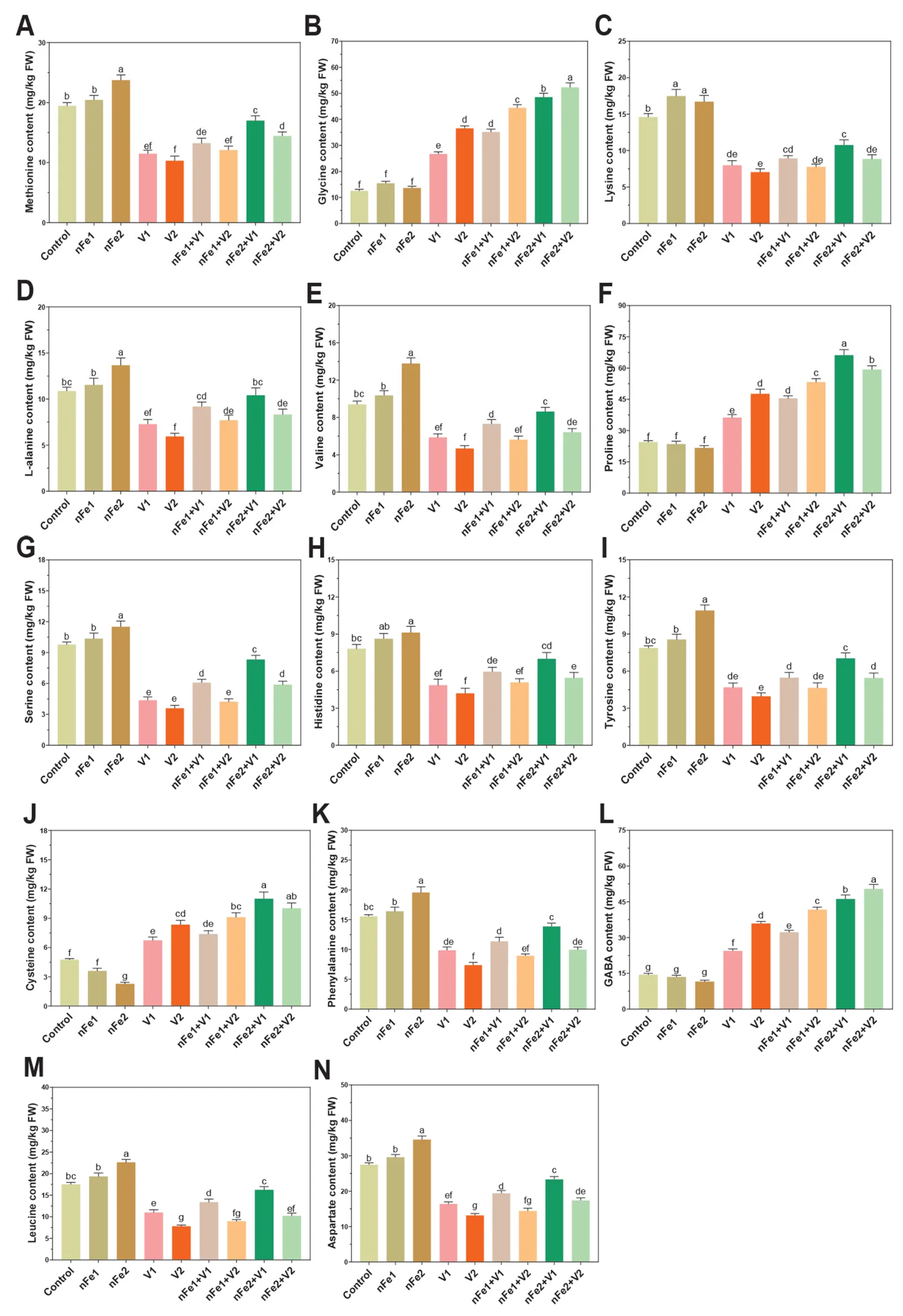
Effects of foliar-sprayed nFe on root amino acid profiling with and without vanadium (V) stress. (**A**) methionine content, (**B**) glycine content, (**C**) lysine content, (**D**) L-alanine, (**E**) valine content, (**F**) proline content, (**G**) serine content, (**H**) histidine content, (**I**) tyrosine content, (**J**) cysteine content, (**K**) phenylalanine content, (**L**) GABA content, (**M**) leucine content, and (**N**) aspartate content. Different lower letters in figures above the bars denote statistically significant differences among treatments (*p* < 0.05, two-way analysis of variance (ANOVA), followed by Tukey’s HSD test).

Exposure to V and nFe significantly modulated the root amino acid (AA) profile, including essential amino acids (EAAs) and non-essential amino acids (NEAAs). The treatments of only V1 and V2 decreased several AAs, *viz*., phenylalanine (Phe) by 38% and 53%, leucine (Leu) by 37% and 56%, valine (Val) by 39% and 49%, histidine (His) by 38% and 47%, methionine (Met) by 40% and 48%, serine (Ser) by 53% and 63%, lysine (Lys) by 45% and 53%, L-alanine (Ala) by 35% and 47%, tyrosine (Tyr) by 40% and 51%, and aspartate (Asp) by 41% and 52%, respectively, compared to the control. On the other hand, some AAs increased, *viz*., cysteine (Cys) by 43% and 76%, glycine (Gly) by 112% and 192%, proline (Pro) by 49% and 94%, and gamma-aminobutyric acid (GABA) by 71% and 149%, under V1 and V2 stress, respectively, compared with the control (Figure 5A–N). 

Supplementation with nFe, especially nFe2, notably modulated the V1- and V2-induced alterations in AAs. The nFe2 + V1 and nFe2 + V2 treatments significantly improved or increased Phe by 41% and 35%, Leu by 48% and 32%, Val by 47% and 37%, His by 44% and 31%, Met by 47% and 39%, Ser by 89% and 65%, Lys by 35% and 27%, Ala by 44% and 40%, Tyr by 50% and 38%, and Asp by 42% and 33%, respectively, compared with corresponding V1 and V2 treatments. Similarly, nFe2 + V1 and nFe2 + V2 further increased the Aas, *viz*., Cys by 64% and 21%, Gly by 81% and 43%, Pro by 83% and 26%, and GABA by 90% and 41%, respectively, compared with the corresponding V1 and V2 treatments. In the current study, EAAs (Val, Met, Phe, Leu, and Lys) were decreased with exposure to V1 and V2, while nFe reversed the trend and improved their levels. The down-regulation in these EAAs by V-only treatment can be associated with a direct impact on N metabolism and amino acid synthesis or degradation pathways, as explained in [38]. The nFe-mediated protection can be attributable to decreased V accumulation and reduced oxidative stress. The reduction in Met content (Figure 5A) was possibly linked to its role as a precursor of S-adenosylmethionine (SAM), an important methyl donor whose metabolism may be altered under metal stress [39]. Some other NEAAs such as Ser, Ala, Tyr, His and Asp (which have been known for HM stress responses) were notably decreased under V stress. Ser is utilized during Cys biosynthesis and plays a crucial role in metal detoxification. His is also a proteinogenic AA and its involvement in metal-stress responses has been established [39]. Tyr is synthesized through the shikimate pathway, which utilizes phosphoenol pyruvate (PEP), a glycolytic intermediate. Recent studies have reported the down-regulation in glycolytic enzymes under metal stress [40]. There is a possibility that the alterations in PEP metabolism and its utilization through the shikimate pathway under V stress may have contributed to reduced Tyr levels in roots [41]. The inclusion of nFe increased the Tyr content toward the level of Tyr content in the control; Tyr also serves as a precursor for different metabolites involved in metal-stress responses [42]. The nFe supply to V-stressed plants enhanced NEAA contents compared with V-only stress and partially restored their level. In the present study, the Pro content increased under V-only treatment and these AAs further increased under nFe + V treatments. Pro not only acts as an osmoregulator but also plays a crucial role in protein and membrane stabilization, antioxidant defense and ROS scavenging. Enhanced Pro levels following NP applications may contribute to improved osmotic adjustments and antioxidant defense, as supported by previous studies [43]. Our results displayed that V stress increased GABA content and nFe treatment further increased GABA levels (Figure 5L). Increased GABA accumulation has been reported in plants exposed to metal stress [44]. Higher GABA levels in *B. juncea* tissues suggest a beneficial role of GABA in C, N and S metabolism and tolerance responses under arsenic stress [45]. A recent study reported that SiNPs enhanced GABA contents under Cd stress conditions, further supporting the involvement of GABA in antioxidant defense and protection against oxidative stress [46].

**Figure 6:**
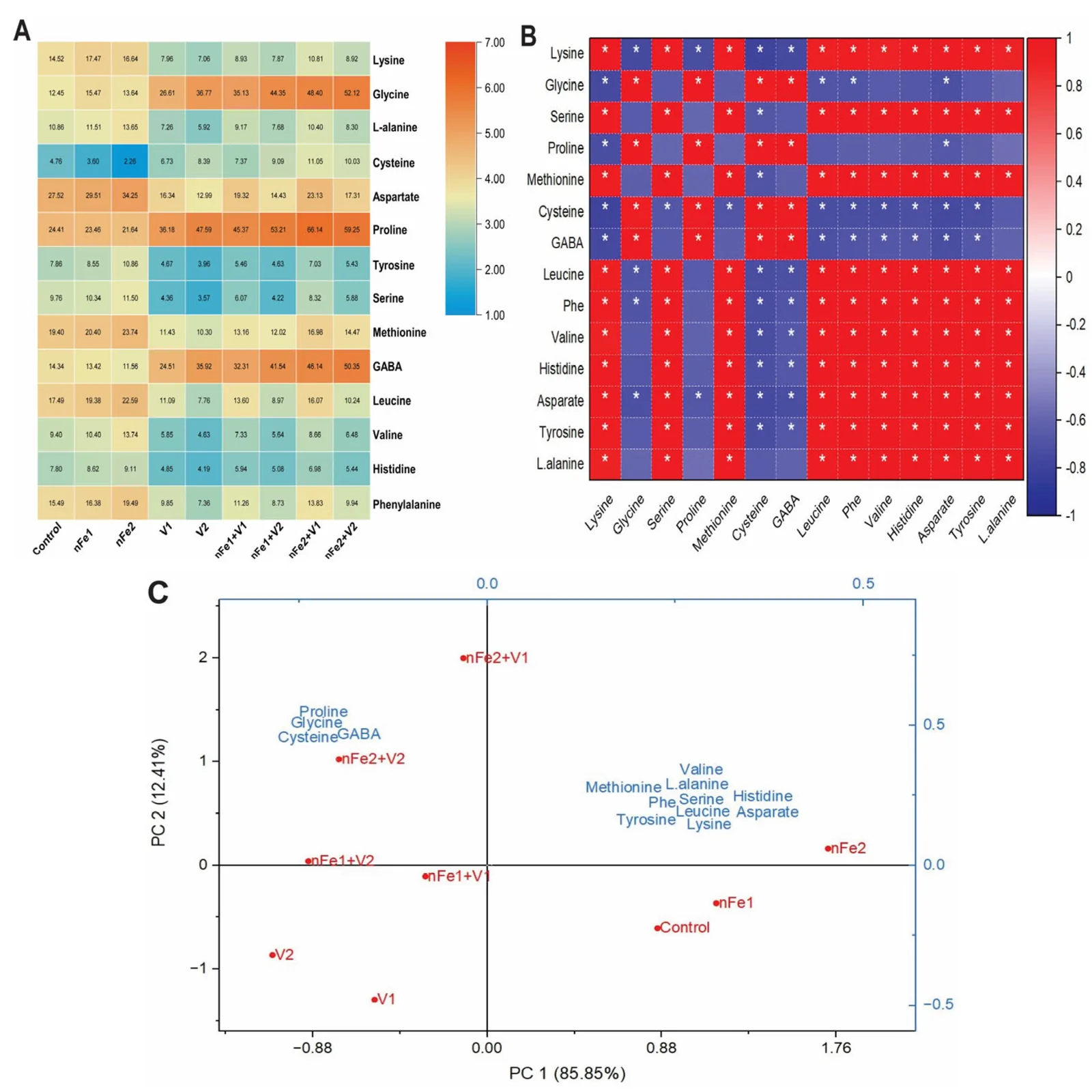
Quantitative heatmap of amino acids (**A**), Pearson correlation matrix (**B**) and principal component analysis biplot (**C**) revealed distinct separation among the applied treatments. The correlation coefficient ranges from −1 (strong negative correlation, as shown in blue) to +1 (strong positive correlation, as shown in red). Significance levels are shown by asterisks (* *p* ≤ 0.05).

A quantitative heatmap of amino acids (A), Pearson correlation matrix (B) and principal component analysis (PCA) biplot (C) revealed the distinct separation among the applied treatments (Figure 6A–C). 

The heatmap demonstrated distinct changes in essential and non-essential amino acid profiles under different treatments (Figure 6A). In particular, the stress-responsive N compounds such as Gly, Pro, GABA and Cys exhibited an increase under V stress and reached their highest levels under combined nFe + V treatments. Conversely, essential (Lys, Val, His, Phe and Met) and non-essential (Asp, Ser, Tyr and Ala) amino acids showed reduced accumulation relative to the control and nFe-only treatments. The preferential distribution of C and N-related metabolites toward Pro, GABA and Cys revealed an active metabolic defense response. This metabolic shift may prioritize ROS scavenging, osmotic adjustment and maintenance of redox balance under V stress. The Pearson correlation matrix revealed clear binary clusters among the targeted amino acids as supported by correlation coefficients (*p* < 0.05) (Figure 6B).

**Figure 7:**
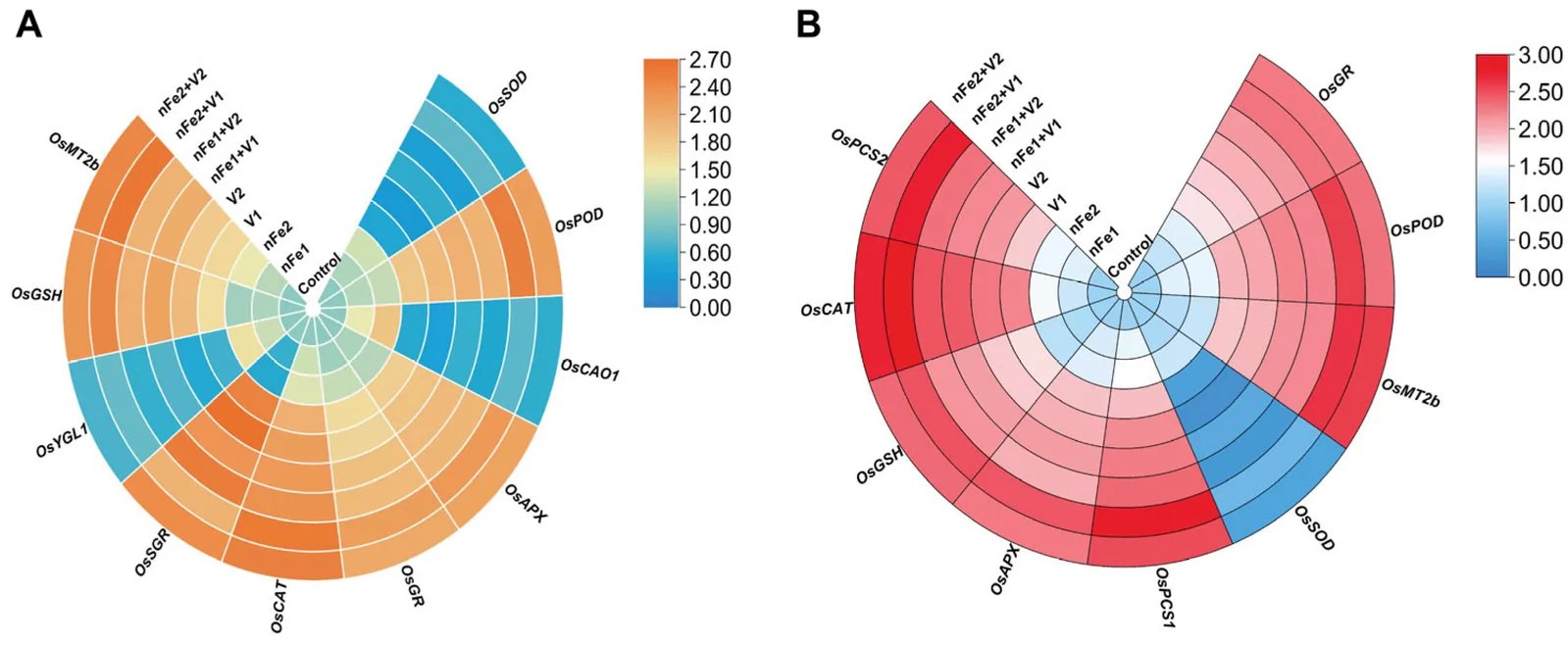
Effects of foliar-sprayed biogenic nFe on the transcription of chlorophyll synthesis (*OsCAO1* and *OsYGL1*) (**A**), chlorophyll degradation (*OsSGR*) (**A**), antioxidant defense system (*OsAPX*, *OsSOD*, *OsPOD*, *OsGR*, and *OsCAT*) (**A**,**B**), and phytochelatin (*OsPCS1, OsMT2b, OsPCS2* and *OsGSH*) (**A**,**B**) responsive genes with and without vanadium (V) stress.

In group 1 (stress-defense cluster), Pro, Cys, Gly and GABA showed strong and significantly positive correlations amongst themselves and strong negative correlations with the remaining AAs. In group 2 (protein synthesis or primary metabolism cluster), Ser, Met, Phe, Val, His, Tyr, Asp, Ala, Leu and Lys form a co-regulatory network and displayed strong positive correlations. Under the applied treatments, a negative cross-correlation between both groups indicates a coordinated metabolic trade-off. These patterns may reflect a preferential accumulation of AAs associated with protective responses such as osmotic adjustments. Principal component 1 (PC 1) accounted for 85.85% and principal component 2 (PC 2) explained 12.41% of total variance (Figure 6C). PC1 clearly separated control and nFe-only treatments (positioned in the positive domain) from V-only and nFe + V treatments (positioned in the negative domain). Vectors for Met, Ser, Tyr, Asp, Val, His, Lys, and Ala were closely aligned with positive PC1, leading to separation of control and nFe treatments. Conversely, loading factors for GABA, Pro, Gly and Cys were projected into the upper left (negative PC1, positive PC2) and strongly drove the division of nFe2 + V1 and nFe2 + V2 treatment groups. Elevated total variance verified by PC1 and PC2 indicates a strong association between the treatment combination and observed amino acid profiles. The transcription profiles of genes related to chlorophyll synthesis, chlorophyll degradation, antioxidant defense, and phytochelatin pathways are depicted in Figure 7A,B.

### 2.7. nFe Protected V-Induced Ultrastructural Damage

**Figure 8:**
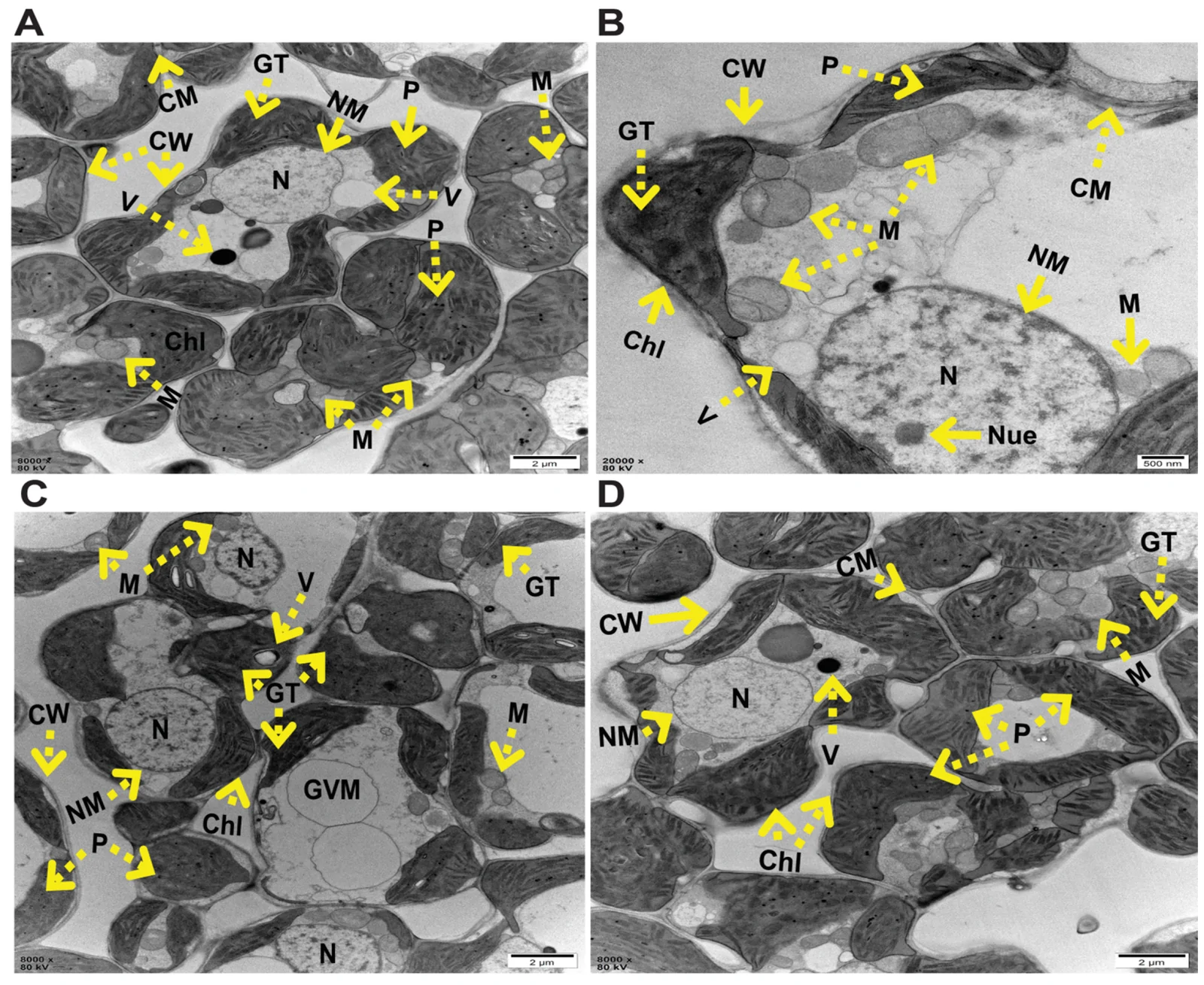
Effects of foliar-sprayed biogenic nFe on the ultra-structural changes in the leaf mesophyll cells treated with and without vanadium (V)-stress treatments: (**A**) control, no-stress condition, (**B**) V2-stress only, (**C**) nFe1 + V2 treatment, and (**D**) nFe2 + V2 treatment. Mitochondria (M), cell wall (CW), cell membrane (CM), chloroplast (Chl), vacuole (V), plastoglubuli (P), nucleolus (Nue), starch grain (SG), nucleus (N), granum thylakoid (GT), giant vacuolar membrane (GVM), and nuclear membrane (NM).

The changes in cellular structures were observed in leaf mesophyll cells from control, V-stressed and nFe + V treated plants (Figure 8A–D). 

In the untreated control, a well-developed cell wall (CW), cell membrane (CM), mitochondria (M), distinct nucleus (N) with a nuclear membrane (NM), clear and large-sized vacuole (V) and number of plastoglobuli (P) adjacent to chloroplasts were noticed (Figure 8A). Under the V2-only treatment, scattered CW with CM, poorly visible N with nucleolus (Nue) and NM, less distinct visible granum thylakoids (GT), small-sized P, and swollen and irregularly shaped M and V were noticed (Figure 8B). Under the nFe1 + V2 treatment, a moderate and partial recovery from cellular damage was observed by improved CW, CM, N, NM, M and Chl structures, organized thylakoid stacks as GT and localized formation of GVM structure (Figure 8C). Under nFe2 + V2 treatment, a significant recovery from V-induced cellular damage was noticed. Mesophyll cells demonstrate well-elongated Chl, compact CW and CM, dense M, well-organized GT with P accumulation, developed N and NM structures and central V that have regained structure (Figure 8D). These observations reveal that nFe mitigated the V-mediated cell damages by recovering structures of thylakoid grana and mitochondrial cristae, which may have helped restore photosynthesis performance and cellular protection against oxidative stress. These findings were supported by previous studies demonstrating that nanomaterials can diminish HM-induced ultrastructural damage to plant tissues [3,36].

### 2.8. Correlation Analysis Partitioned the Effects of nFe on O. sativa Plants Under V Stress

**Figure 9:**
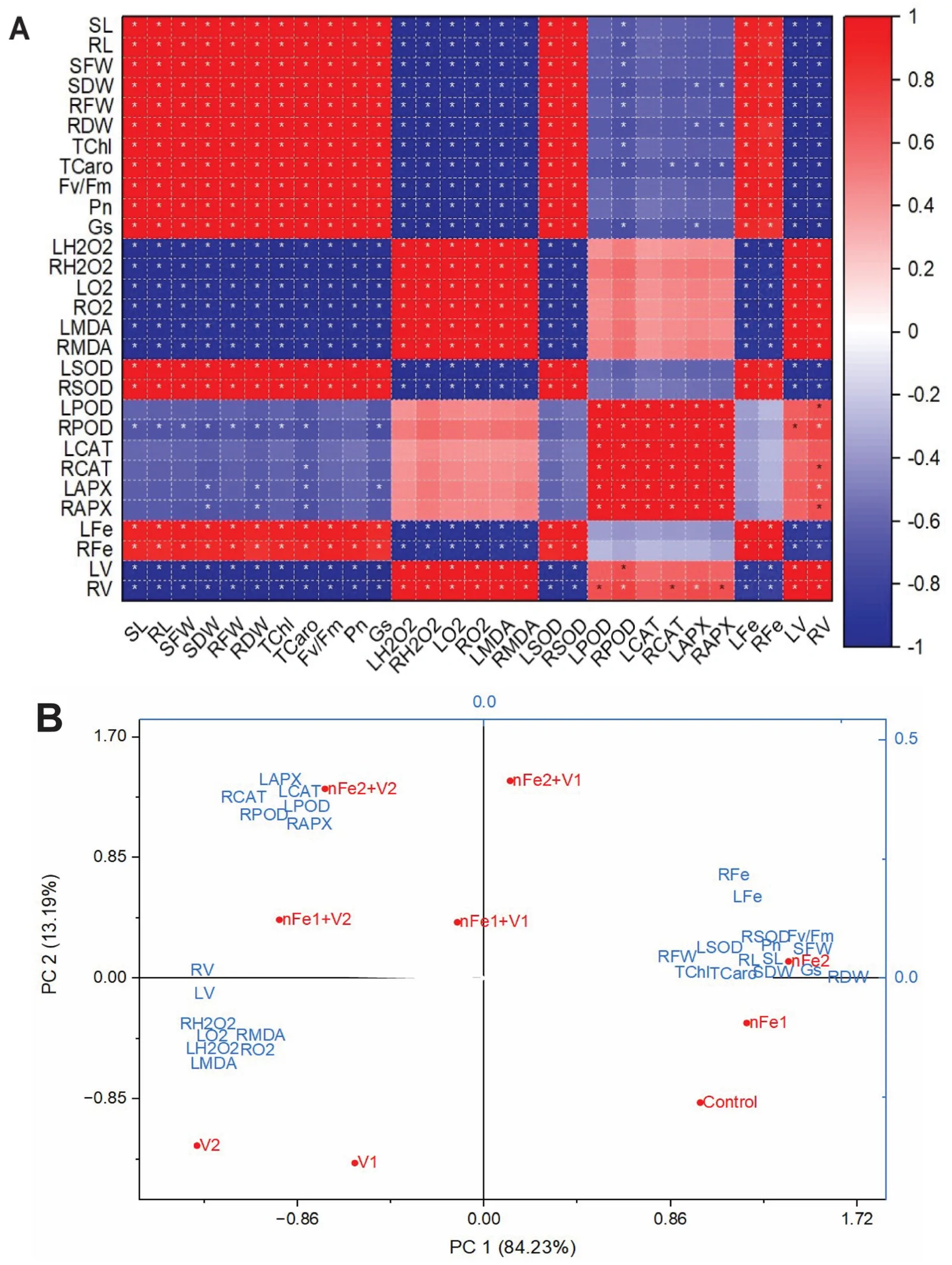
Correlation analysis of applied treatments (**A**) and Principle component analysis (PCA) (**B**) against different studied parameters. Correlation analysis of studied parameters against different treatments was performed. The correlation coefficient ranges from strong negative correlation (denoted as −1) to strong positive correlation (denoted as +1). Significant levels are denoted by asterisks (* *p* ≤ 0.05). Vector arrows represent the contribution of each variable to PCA. The direction and length of the vector arrow display the impact of each variable on the corresponding treatment. PCA biplot illustrating the relationship between nano-iron (nFe) alone treatments, vanadium (V) stress levels, and various physiological, biochemical, and growth parameters. PC1 and PC2 account for 84.23% and 13.19% of the total variance, respectively.

A correlation analysis was performed among the various variables in leaves and roots (Figure 9A). The Pearson correlation matrix distinguished the observed traits into distinct (positive and negative) and co-regulated modules as noticed by statistical significance (*p* < 0.05). Morphological traits (SL, RL, LFW, RFW, LDW, and RDW), photosynthesis-related traits (TChl, TCaro, Fv/Fm, Gs and Pn), and Fe contents (LFe and RFe) exhibited strong and statistically significant positive correlations (red box). Oxidative stress markers (H_2_O_2_, O_2_^•–^ and MDA) and V level in leaves and roots were linked with a positive cluster that displays significant negative correlation with plant growth, photosynthesis and SOD activity.

**Figure 10:**
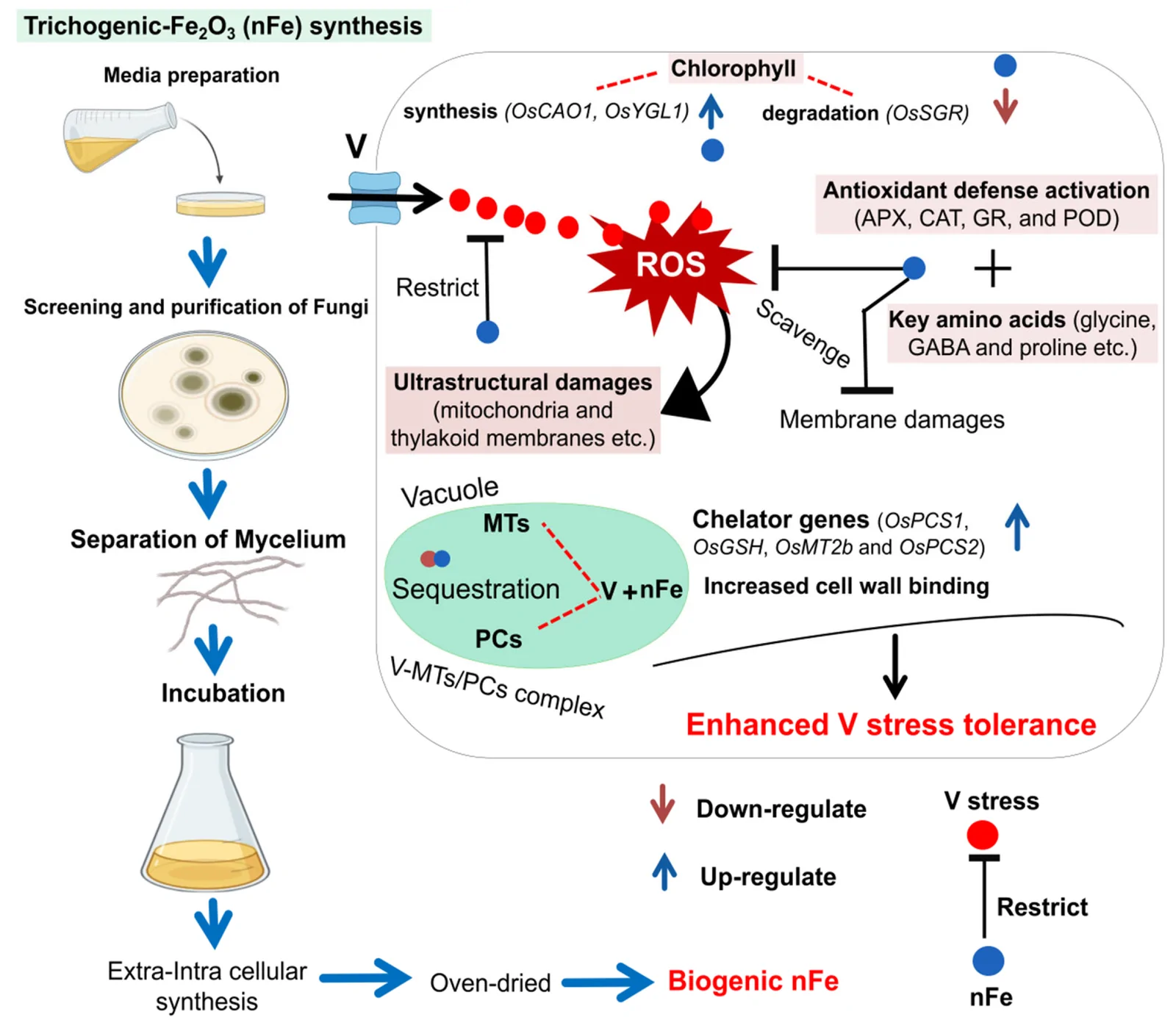
Schematic diagram illustrating the V-stress tolerance mechanisms mediated by biogenic nFe. The left panel outlines the green synthesis workflow of biogenic nFe. Upon root uptake, excessive V triggers reactive oxygen species (ROS), causing oxidative stress and damaging leaf ultrastructures. Conversely, nFe promotes chlorophyll synthesis while protecting its degradation, thereby enhancing photosynthetic performance. The synergistic effects of antioxidant defense-related enzymes and amino acids play crucial roles in scavenging ROS and activating plant defense responses. Furthermore, nFe regulates the V-subcellular distribution by promoting its cell wall binding, restoring vacuolar compartmentalization, enhancing V chelation via phytochelatins (PCs) and metallathioneins (MTs), and restricting toxic ion entry into cellular organelles, collectively mitigating V toxicity.

Antioxidant enzyme (POD, CAT and APX) activities exhibited positive regulation with one another and showed a negative relationship with stress markers. This module reflects clear physiological trade-off under V-stress conditions. The positive alignment of nFe and SOD activity with growth attributes indicates Fe-homeostasis and SOD defense is critical in protecting photosynthetic apparatus and preserving plant biomass. Principal component 1 (PC 1) accounts for 84.23% and principal component 2 (PC 2) explains 13.19% of the total variance (Figure 9B). PC1 (stress-mitigation route) clearly separates V-stressed treatment (located in negative domain) and nFe treatments (especially nFe2) located in the far positive PC1 domain. Plant morphological parameters, photosynthetic parameters, SOD activity and nFe (leaf or root) were strongly found in the lower quadrant (left) and aligned with V treatments. In contrast, enzymes (POD, CAT and APX) in both leaves and roots were projected into the upper quadrant (left) and denoted as negative PC1 and positive PC2, aligning with treatment combinations. The higher proportion of variance exhibited by PC1 verified that V toxicity and nFe supply are crucial factors related to physiological variation. Overall, nFe application results in a metabolic shift away from V-stress responses, a shift in the ROS quadrant toward the positive PC1 domain and is largely associated with higher Fe accumulation, enhanced antioxidant defense, improved photosynthetic machinery and greater biomass recovery.To summarize the overall research, a schematic diagram was created to highlight the mechanisms underlying nFe-mediated V stress tolerance (Figure 10).

## 3. Materials and Methods

### 3.1. Characterization Data of Biologically Synthesized Iron Oxide Nanoparticles (nFe)

The *trichoderma*-mediated mycosynthesis of nFe was achieved by following the established protocol [47] and further characterization details are provided in Figure S1 and Text S1. The stability and hydrodynamic behavior of nFe was evaluated before treatment application. This biogenic capping provides surface charge stabilization and steric hindrance, notably minimizing the spontaneous aggregation in liquid media compared to uncapped chemical NPs [47]. *Trichoderma* was chosen due to robust secretion of extracellular enzymes and biomolecules, enabling a simple, scalable, and non-toxic synthesis without requiring complex intracellular extraction steps. Compared to other biogenic sources, *Trichoderma*-derived NPs exhibit organic capping layers that impart superior colloidal stability, exhibit low aggregation, and deliver biostimulatory as well as defense-priming mechanisms [48].

### 3.2. Plant Materials, Growth Conditions and Applied Treatments

The mature seeds of rice cultivar “Qian You 1” were procured from the Seed Science Center, Zhejiang University, Hangzhou, China. Prior to sowing, seeds were sterilized with 0.5% sodium hypochlorite for 5 min and thoroughly washed four times using deionized water. For seed emergence, sterilized seeds were soaked in deionized water and put in an incubator in the dark at 27 °C for three days. Germinated seeds were then shifted to plastic-net floating plates placed over boxes containing half-strength nutrient solution and placed in a controlled growth chamber for further plant growth. The conditions of the growth chamber were kept at a 16 h light/8 h dark photoperiod, day/night temperature of 28/23 °C, light intensity of 800–1000 μmol m^−2^ s^−1^ and relative humidity of 60 ± 3%. Half-strength nutrient solution (pH 5.7) was used for the first three days and then switched to full-strength until the 21st day of transplantation. At the two-leaf stage, uniform sized seedlings were treated with V stress using NH_4_VO_3_ (ammonium metavanadate) at 10 mg/L (V1) and 20 mg/L (V2) concentrations in nutrient solution. The suspensions of nFe were prepared to achieve the targeted concentrations of 3 mg/L (nFe1) and 6 mg/L (nFe2). Stock suspensions of nFe were initially prepared by dispersing the weight mass of powdered NPs suspended in double-distilled water. To ensure uniform dispersion and prevent agglomeration, stock suspensions were subjected to sonication (using ultrasonic agitation) at 40 kHz for 30 min. From this stock suspension, working concentrations of nFe1 and nFe2 were prepared through serial dilution using double-distilled water. Prior to treatment application, solutions were re-sonicated to maintain uniform and stable NP distribution. On a daily basis, foliar treatments of nFe at the two-leaf stage were performed using a handheld atomizer and each plant was evenly sprayed until droplet run-off formed on the leaf surfaces. The nutrient solution was refreshed every 48 h. The applied nine treatments were: (1) control (CK), nutrient-only solution; (2) 3 mg/L nFe as nFe1; (3) 6 mg/L nFe as nFe2; (4) 10 mg/L V as V1; (5) 20 mg/L V as V2; (6) 3 mg/L nFe as nFe1 + 10 mg/L V as V1; (7) 3 mg/L nFe as nFe1 + 20 mg/L V as V2; (8) 6 mg/L nFe as nFe2 + 10 mg/L V as V1; and (9) 6 mg/L nFe as nFe2 + 20 mg/L V as V2. The applied V concentrations were chosen based on the screening experiment (Figure S2). All applied treatments were performed in at least four biological replicates per treatment (each individual pot as an independent unit) and four technical replicates derived from the separate leaves. The experiment was arranged in completely randomized design. After 7 days of treatment (21 days after transplantation), plant materials were thoroughly water-washed and biomasses were recorded. Some fresh plant materials (leaves and roots) were directly taken for the morphological, physiological and cellular parameters. The remaining plant materials were quickly stored in liquid N at −80 °C for biochemical and molecular analysis.

### 3.3. Measurement of Morphological Parameters, Biomass and Photosynthesis Phenomics

Shoot and root lengths were measured manually. The fresh biomass of shoot and root (g/seedling) were manually measured, after which the samples were dried at 65 °C for the estimation of dry biomass. The second fully expanded leaves were selected for spectrophotometric quantification of total chlorophyll and total carotenoids as described previously [49]. Leaf net photosynthetic rate and stomatal conductance was measured between 9 and 12am under light intensity of 1000 to 1500 µmolm^−2^s^−1^ using an LI-COR 6400 photosynthesis system (LI-COR Inc., Lincoln, NE, USA). The second fully expanded leaves from each treatment were kept in dark adaptation for 30 min, and then leaves were exposed toan imaging pulse-amplitude modulated (PAM) fluorimeter (IMAG-MAXI; Heinz Walz, Effeltrich, Germany) for the measurement of maximal quantum yield of PSII as *Fv/Fm* [3].

### 3.4. Quantification of Hydrogen Peroxide (H_2_O_2_) and Superoxide (O_2_^•−^) Along with Their Histochemical Analysis via Root Staining, and Malondialdehyde (MDA) Contents

For the quantification of H_2_O_2_, plant tissues (0.5 g) were homogenized in 0.15% (*w*/*v*) TCA in a cold bath, followed by centrifugation at 15,000 *g* for 15 min. The resultant 0.5 mL supernatant was mixed with 0.5 mL of 50 mM potassium phosphate buffer and 1 mL of 1 M KI solution. The H_2_O_2_ and O_2_^•−^ contents in plant tissues were determined following the established protocol [50]. The accumulation of H_2_O_2_ and O_2_^•−^ in root tip cells was observed with 3,3-diaminobenzidine as DAB and nitroblue tetrazolium as NBT for root staining [51]. Lipid peroxidation was assessed by measuring MDA contents, according to the Heath and Packer method [52].

### 3.5. Assays of Antioxidant Enzyme Activities

Approximately 0.5 g plant sample was ground in 50 mM potassium phosphate buffer and centrifuged at 12,000 *g* for 15 min at 4 °C. The resulting supernatant was further utilized in assays for subsequent antioxidant enzymes. The following established methods were utilized to assay superoxide dismutase (SOD), peroxidase (POD) [53] and catalase (CAT) [54], ascorbate peroxidase (APX) [55] and glutathione reductase (GR) [56]. Glutathione content in plant tissues was determined by following an established method [57]. The estimation of metallothionein protein in leaf and root tissues was determined by following the previous method [58].

### 3.6. Quantification of Amino Acid Profiling and Isolation of Subcellular Components

The quantification of amino acids (AAs) in rice roots was carried out using the pico tag method as explained in [59]. The subcellular distributions of V in the cell wall, soluble fractions, mitochondria, plastid or chloroplast, and nucleus were determined by following the earlier method [60] with minor changes. Fresh leaf and root samples (0.5 g) were pulverized in liquid N. The obtained powder was homogenized in pre-chilled buffer (50 mM Tris-HCl (pH 7.5), 5 mM ascorbic acid, 0.25 M sucrose and 1 mM dithioerythritol), followed by a gradient centrifugation method. The resulting homogenate was later centrifuged at 300 to 500 *g* for 5 min and sediments were denoted as cell wall fractions. The supernatant was further centrifuged at 2000 *g* for 10 min and regarded as a plastid or chloroplast fraction. The supernatant was centrifuged at 5000 *g* for 30 min and the deposition was referred to as nucleus. For the fourth centrifugation step, the supernatant was centrifuged at 10,000 *g* for 20 min and the deposition was referred to as the mitochondrial fraction. The remaining and last supernatant was considered as the soluble fraction (vacuolar component). After the separation technique, each subcellular pellet was digested and the V concentration in each subcellular component was quantified using ICP-MS (NexION 300X, PerkinElmer, Shelton, CT, USA).

### 3.7. Measurement of Fe and V Content, and Observation of Cellular Structures

Dried leaf and root samples (0.5 g each) were crushed and digested with a 6 mL mixture of H_2_O_2_ (1 mL) and concentrated HNO_3_ (5 mL) (1:5, *v*/*v*) using a microwave digestion system. The final Fe concentration was determined using inductively coupled plasma optical emission spectrometry (ICPS-7500, Shimadzu, Kyoto, Japan) [51], whereas V content was estimated using graphite furnace atomic absorption spectrophotometry (GFAAS-GTA 120) [61]. Quality control for V content was conducted by reference materials, GBW07603 (GSV-2) (bush branches and leaves) from center for standard reference, Chinawith error ranges of +10% to −10% (reflects data accuracy). Samples for transmission electron microscopy (TEM, H-7650; Hitachi, Ibaraki, Japan) analysis were prepared by following established protocols [3].

### 3.8. Total RNA Extraction and Quantitative Real-Time PCR (qRT-PCR) Assays of Targeted Genes

The isolation of total RNA from leaves and roots was performed with an RNA isolation kit (Takara, Kyoto, Japan). Genomic DNA contamination was emitted with a Prime-Script^TM^ reagent and gDNA eraser kit (Takara, Co., Ltd., Kyoto, Japan). Next, RNA quality and quantity were evaluated using a Nano Drop-1000 spectrophotometer (Thermo Scientific, Waltham, MA, USA). cDNA was synthesized from 1 μg of high-quality RNA using a PrimeScript^TM^ RT reagent kit. qRT-PCR was conducted (triplicate technical replicate of each biological replicate) using the SYBR^®^ Premix Ex Taq II on CFX96^®^ Real-Time PCR System (Bio-Rad, Hercules, CA, USA). *OsActin* was used as an internal control gene and relative gene expression was quantified via comparative Ct method [62]. Gene-specific primer sequences utilized in qRT-PCR are given in the Supplementary Data (Table S1).

### 3.9. Statistical Analysis

Statistical analysis was performed using SPSS 25.0 (IBM Corp., Armonk, NY, USA). To differentiate among treatment groups, a two-way analysis of variance (ANOVA) was conducted, followed by Tukey’s HSD test at *p* < 0.05. All values are reported as mean ± standard deviation. Graph Pad Prism (version 8.0) was used to produce graphs.

## 4. Conclusions

This study demonstrates the significant potential of *trichoderma*-synthesized nFe as an innovative approach to mitigate the V toxicity in rice seedlings. Under V-only stress, the excessive accumulation of V ions disrupted growth traits, photosynthetic performance, redox balance (by inducing oxidative stress and impairing the antioxidant defense system), V subcellular distribution and membrane stability, modulated amino acid (essential and non-essential) metabolism, and damaged leaf mesophyll cells. The application of nFe significantly minimized the V accumulation in plant tissues, enhanced V retention in the cell wall fraction, improved photosynthesis, and rescued the cell from damage by lowering oxidative stress. Moreover, nFe improved the antioxidant defense system and related gene expression, Fe-assimilation ability, and amino acid levels, and ultimately improved plant tolerance and growth. This study not only provides a deeper understanding of the physio-metabolic mechanisms underlying V-stress resilience at the seedling stage, but also provides a practical application for nFe as a potential nano-fertilizer to optimize rice cultivation under V-contaminated conditions.

## Data Availability

All data generated or analyzed during this study are included in this published article and its Supplementary Information files.

## Supplementary Materials

The following supporting information can be downloaded at https://www.mdpi.com/article/10.3390/ijms27219588/s1.
