# Supplementary material for: Trichoderma-Synthesized Iron Oxide Nanoparticles Confer Vanadium Tolerance in Rice Through Multiple Defense Responses
Source: Int J Mol Sci. 2026 Oct 27;27(21):9588. doi: 10.3390/ijms27219588 (PMC13763529; doi:10.3390/ijms27219588)
Supplement: Supplementary file 1 [file ijms-27-09588-s001.zip › ijms-4605268-supplementary.pdf]

Supplementary Material

Article

Trichoderma-Synthesized Iron Oxide Nanoparticles confer Vanadium Tolerance in Rice through Multiple Defense Responses

Hafsah Shahbaz [1], Zaid Ulhassan [2,*], Zainab Shahbaz [3], Zohaib Kaleem [4], Aamir Mehmood Shah [5], Afrah E. Mohammed [6], Modhi O. Alotaibi [7], Dalal H. Alkhalifa [7], and Ali El-Keblawy[8,9]

[1] Institute of Animal and Dairy Sciences, University of Agriculture Faisalabad, Faisalabad 38040, Pakistan; hafsahshahbaz25@gmail.com (H.S.)
[2] School of Breeding and Multiplication (Sanya Institute of Breeding and Multiplication), Hainan University, Sanya 572000, China; 997153@hainanu.edu.cn (Z.U.)
[3] Key Laboratory of Forest Pathology, School of Forestry, Northeast Forestry University, Harbin 150040, China; zainabshahbaz78@gmail.com (Z.S.)
[4] College of Life Sciences and Oceanography, Shenzhen University, Shenzhen 518000, China; naarzadazohaib6051@gmail.com (Z.K.)
[5] College of Landscape Architecture, Sichuan Agricultural University, Chengdu, Sichuan 611130, China; aamirmehmood55@hotmail.com (A.S.)
[6] Microbiology and Immunology Unit, Natural and Health Sciences Research Center, Princess Nourah bint Abdulrahman University, Riyadh, Saudi Arabia; AFAMohammed@pnu.edu.sa (A.M.)
[7] Department of Biology, College of Science, Princess Nourah bint Abdulrahman University, P.O. Box 84428, Riyadh 11671, Saudi Arabia; mouotaebe@pnu.edu.sa (M.A.), dhalkalifah@pnu.edu.sa (D.A.)
[8] Department of Applied Biology, College of Sciences, University of Sharjah, PO Box 27272, Sharjah, UAE.
[9] Faculty of Pharmacy, Alsalam University, Tanta, Egypt; akeblawy@sharjah.ac.ae (A.E.)
* Correspondence: 997153@hainanu.edu.cn

Table of Content **Page number**

Text S1 Biogenic synthesis and characterization of biologically-synthesized Fe_2O_3 NPs (nFe)…………..2
Text S2 Screening experiment for the selection of vanadium stress…………………..…………………..…2
Figure S1 Characterization of biogenic Fe_2O_3NPs (nFe). (A) scanning electron microscopy; (B) transmission electron microscopy; (C–F) elemental composition of nFe………………………..….…..…3
Figure S2 Impacts of different vanadium levels on rice height and biomass (fresh and dry), and SPAD value……………………………..……………………………………………………………………….....4
Table S1 Oligonucleotide primer sequences used for qRT-PCR analysis……………………………..…5

Materials and methods

Text S1 Biogenic synthesis and characterization of biologically-synthesized Fe_2O_3 NPs (nFe)

The trichoderma-mediated mycosynthesis of nFe was achieved by following the established protocol [1]. The biosynthesized nFe was analyzed for morphology and optical characteristics via multiple analytical techniques. To ensure reproducibility and accuracy in results, all the analysis was performed in triplicates. The specific shape and size of fungal mediated nFe were determined by TEM, a JEOL JEM-1230 instrument (JEOL Ltd., Akishima, Japan). The distribution of nFe size was analyzed by SEM, a Hitachi TM-1000 instrument (Hitachi Ltd., Tokyo, Japan). Moreover, the composition of elements and richness of Fe and O in Fe_2O_3 NPs specimen was examined using EDS and detector-equipped FE-SEM. The biogenic nFe were synthesized in a single, large-scale independent batch (about 10 g) to ensure maximum consistency across all experimental treatments and replicates. This approach was intentionally chosen to eliminate the 'batch effect' as a confounding variable in the plant physiological responses.

Text S2 Screening experiment for the selection of vanadium stress

After sterilization with 5% sodium hypochlorite solution for 8-10 min, rice seeds were rinsed with DI water and place in growth media for germination. On the 10th day, germinated seeds were transplanted into plastic containers and further cultivation was performed in greenhouse with day/night temperature of about 33/28°C. On 15th day, plants were treated with different levels of V solution (0 as blank control, 2, 5, 10, 20, 30, 40, 50, and 100 mg/L). Each treatment was repeated six times and four plants were kept in each replicate. Seedlings were harvested on 25th day after 7 days of V treatment during which each treatment was reapplied every 3rd day. Then plant height, plant fresh weight, plant dry weight and chlorophyll content as SPAD value were recorded.

Results and discussion

3.1. Characterization data for biologically synthesized nFe

SEM analysis of the fungal-mediated synthesized nFe displayed least agglomeration and spherical morphology (Figure S1,A). TEM micrographs further confirmed the average size of 25-60 nm and uniform size distribution (Figure S1, B). EDX (Energy-dispersive X-ray) microanalysis confirmed the chemical compositions of the target specimens, as indicated by the prominent peaks (strong signal) for iron (Fe) and oxygen (O). EDX elemental mapping verified that O and Fe were the primary constituents in nFe sample (Figure S1,C–F). These characterization evidence high purity and structural integrity of nFe used in this study.

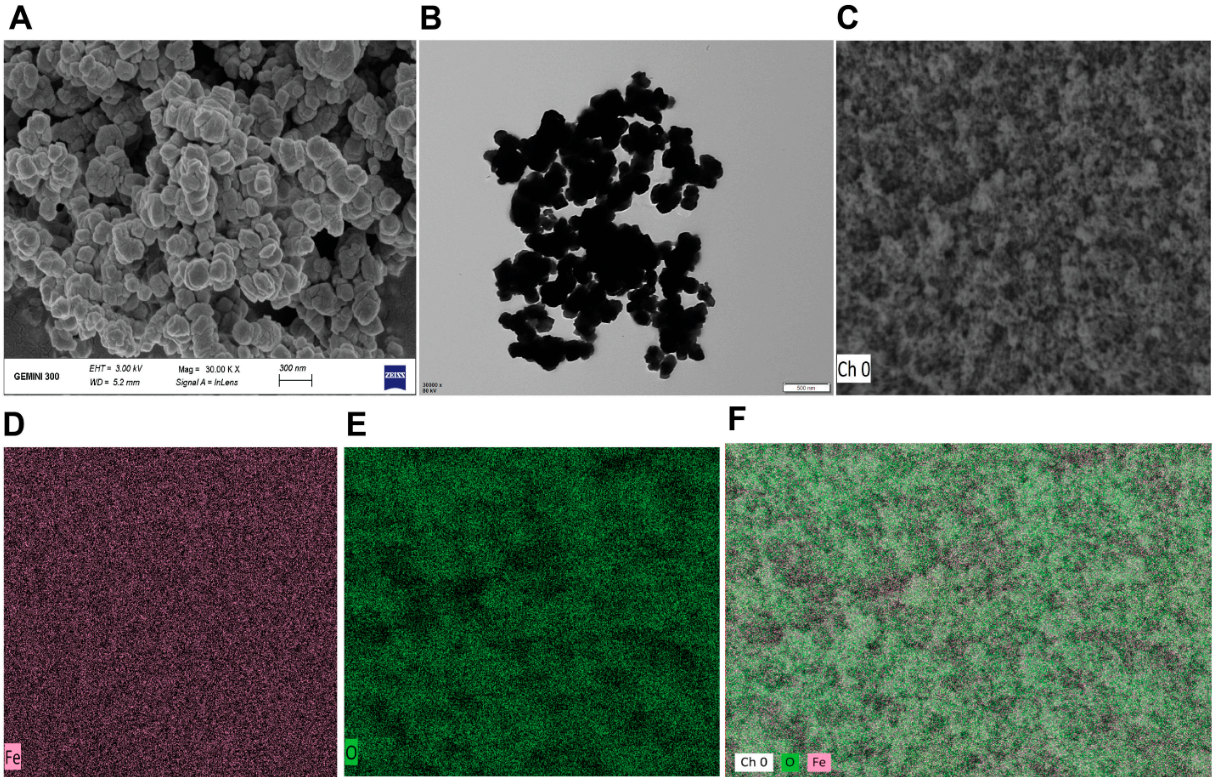
A
B
C
GEMINI 300
EHT = 3.00 kV
WD = 5.2 mm
Mag = 30.00 K X
Signal A = InLens
300 nm
ZEISS
Ch 0
D
E
F
Fe
O
Ch 0
O
Fe

Figure S1

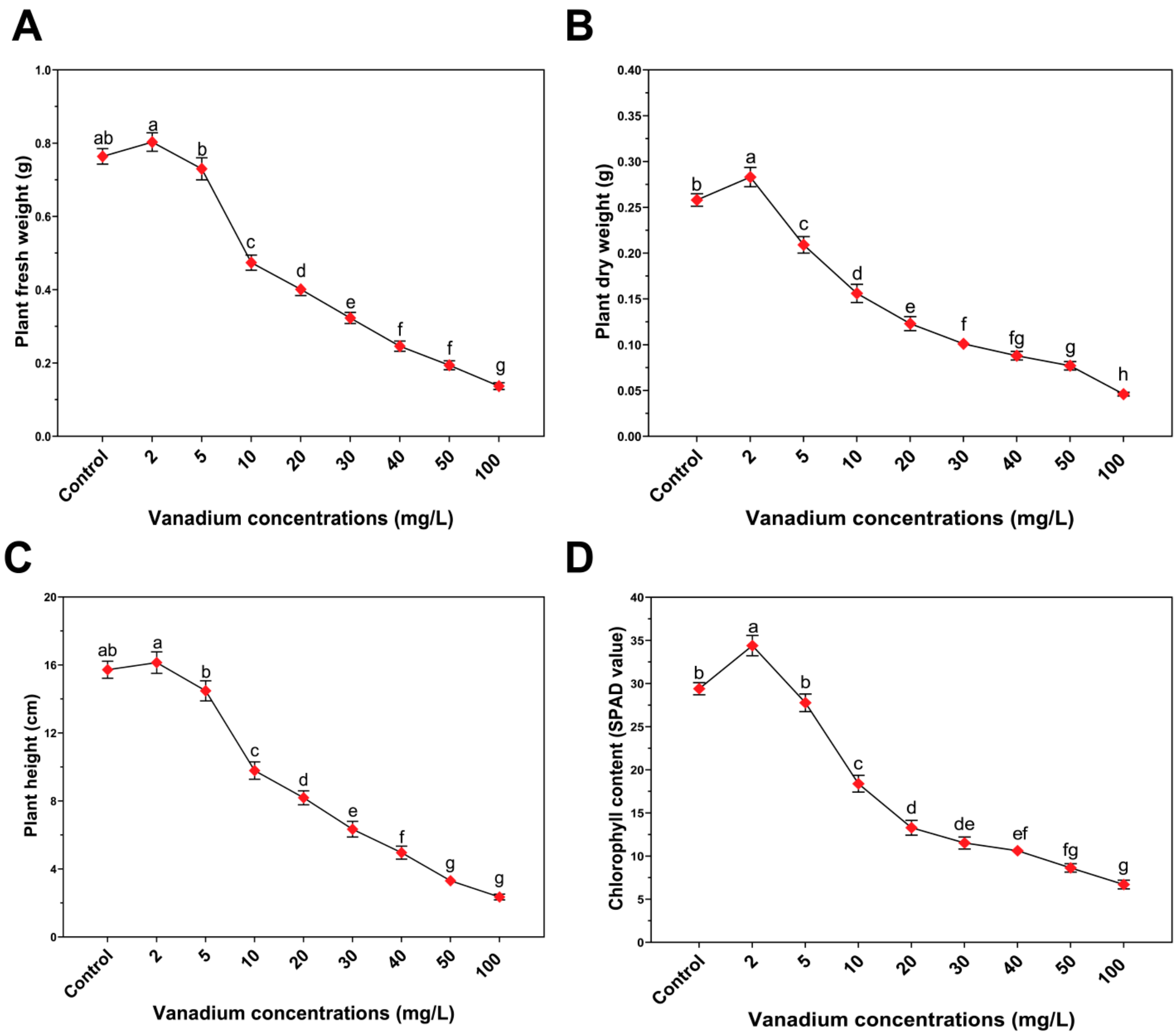
A
Plant fresh weight (g)
1.0
0.8
0.6
0.4
0.2
0.0
ab
a
b
c
d
e
f
f
g
Control
2
5
10
20
30
40
50
100
Vanadium concentrations (mg/L)
B
Plant dry weight (g)
0.40
0.35
0.30
0.25
0.20
0.15
0.10
0.05
0.00
b
a
c
d
e
f
fg
g
h
Control
2
5
10
20
30
40
50
100
Vanadium concentrations (mg/L)
C
Plant height (cm)
20
16
12
8
4
0
ab
a
b
c
d
e
f
g
g
Control
2
5
10
20
30
40
50
100
Vanadium concentrations (mg/L)
D
Chlorophyll content (SPAD value)
40
35
30
25
20
15
10
5
0
b
a
b
c
d
de
ef
fg
g
Control
2
5
10
20
30
40
50
100
Vanadium concentrations (mg/L)

Figure S2

Table S1 Oligonucleotide primer sequences used for qRT-PCR analysis.

Gene name	Primer sequences (5' →3')	
	Forward sequence	Reverse sequence
OsActin	GCTAAGCCAAGAGGAGCT	CTTTGTCCACGCTAATGAAG
OsPOD	GTCCGCAAGATCAGCGACTT	CGGAGTGGAAGGTGACGAT
OsCAT	TGATTGCCAAGGAGAACAACT	TCTCTTGATGAACCGGTCTTG
OsSOD	CACCACCAATGGTTGCATGT	TGGCGGGTCTCATCTTCTG
OsCAO1	GATCCATACCCGATCGACAT	CGAGAGACATCCGGTAGAGC
OsYGL1	TCTTGGTGCGAGCTACATTG	GCTTGCCTGAACTGAAAAGG
OsSGR	AGGGGTGGTACAACAAGCTG	GCTCCTTGCGGAAGATGTAG
OsAPX	TCAAGGACCAACTTCCCATC	AGGGTGTGACCACCAGAAAG
OsGR	AAGTGATTCCTCGCGCTAAA	CTCGTCCTTGAGCATCTTCC
OsMT2b	AAGAAGCCTGGCACGCATGAG	TGCGTGTGTCGATCAATGTTGGA
OsPCS2	CAGGGGGTTCATGCTTATCT	GGCAGGAAGGGATTTCACTA
OsPCS1	TCGCTTCAAATACCCTCCTC	TTTACTTGGGCTGGATCCTC
OsGSH	GGAGCCTATTTGCGGAACA	TGGTATGTAGTATCACCTCATCTGT

References

1. F. Liaquat, A. Alyan, A. Malik, L. Qunlu, U. Haroon, S. ur Rehman, et al., *Trichoderma-mediated iron oxide nanoproduct improves drought tolerance and carbon sequestration in industrial woody plants.* Plant Stress, 2025: p. 100992.
